# A scoping review on the nature and impact of gender based violence on women primary producers

**DOI:** 10.1186/s12905-024-03228-3

**Published:** 2024-07-09

**Authors:** Cathy O’Mullan, Saba Sinai, Sabitra Kaphle

**Affiliations:** CQ University, Bruce Highway, Rockhampton, Queensland Q 4670 Australia

**Keywords:** Gender based violence, Agriculture, Low income countries, Women producers

## Abstract

**Background:**

Women in low- and middle-income countries (LMICs) are primary producers of subsistence food and significant contributors to the agricultural economy. Gender Based Violence (GBV) adversely impacts their capacity to contribute and sustain their families and undermines social, economic, and human capital. Addressing GBV, therefore, is critical to creating safe and inclusive environments for women as primary producers to participate fully in rural communities. The aim of this scoping review is to explore the existing evidence on GBV in the context of women primary producers in LMICs to inform research gaps and priorities.

**Methods:**

A scoping review was conducted using PubMed, Web of Science, Ebscohost and Google Scholar using keywords related to GBV and women producers in LMICs. Peer-reviewed journal articles published between January 2012 and June 2022 were included in the review. Duplicates were removed, titles and abstracts were screened, and characteristics and main results of included studies were recorded in a data charting form. A total of 579 records were identified, of which 49 studies were eligible for inclusion in this study.

**Results:**

Five major themes were identified from our analysis: (1) extent and nature of GBV, (2) the impact of GBV on agricultural/primary production livelihood activities, (3) sociocultural beliefs, practices, and attitudes, (4) aggravating or protective factors, and (5) GBV interventions. Addressing GBV in agriculture requires inclusive research approaches and targeted interventions to empower women producers, promote gender equality, enhance agricultural productivity, and contribute to broader societal development. Despite attempts by researchers to delve into this issue, the pervasive under-reporting of GBV remains a challenge. The true extent and nature of GBV perpetrated against women is far from fully understood in this context.

**Conclusion:**

Despite the significant challenges posed by GBV to the health, economy and livelihoods of women primary producers in LMICs, there is a paucity in the current state of knowledge. To make meaningful progress, more research is required to understand the relationship between GBV and agricultural settings, and to gain nuanced insight into the nature and impact of GBV on women primary producers in different regions and contexts.

## Background

Gender-based violence (GBV) is a widespread public health issue that affects people of all genders, but disproportionately impacts women and girls [1]. GBV is deeply rooted in gender inequality, and is reinforced by patriarchal norms, discriminatory laws, and socio-cultural practice that violate women’s rights [2]. Although GBV can affect individuals from all backgrounds, women living in low- and middle-income countries (LMICs) experience a disproportionate burden due to a range of complex socio-economic and cultural factors [3].

Given women have a significant representation in the agricultural workforce in LMICs and substantial contributions to food and crop production, the impacts of GBV have far-reaching consequences on agricultural productivity and food security [4]. In the context of ongoing climate change, the urgency to address GBV within agricultural settings becomes even more pronounced as climate shocks create a strain on food production and exacerbate food insecurity [5]. Women and girls are often the first to be negatively impacted by increased food insecurity which tends to lead to an increased incidence of violence against women [5, 6]. While GBV is a highly concerning issue gaining increasing attention, particularly within the public health and humanitarian fields, the relationship between GBV and agriculture lacks focus and recognition.

GBV, as defined by UN Women, refers to “harmful acts directed at an individual or a group of individuals based on their gender” [7]. While there is debate over which forms of violence fall under the GBV umbrella [8], for the purposes of this review, we include physical, sexual, economic, and psychological violence. Gender is a product of social and cultural influences rather than an innate characteristic [9], and GBV is intricately linked to the social constructs of gender. Deeply ingrained patriarchy and harmful beliefs and stereotypes about masculinity and femininity shape the roles of men and women, exerting significant power dynamics that perpetuate oppression and gender inequalities [10, 11].

Cultural practices, attitudes and traditions that contribute to GBV in LMICs, such as forced marriage, bride price and female genital mutilation, originate from systemic gender inequality, coercive control, and harmful social norms [12–14]. Although GBV affects individuals of all genders, women experience heightened risks due to gender-based power inequalities and discriminatory laws perpetuated by social norms and practices [12]. Global perspectives on GBV recognize the phenomenon is not incidental or indicative of a woman’s vulnerability; rather it is “embedded in structural systems, norms and long-standing discrimination” [15].

According to the World Health Organization, around one in three women worldwide experience physical or sexual violence from an intimate partner at some point in their lives [1]. Women in LMICs are disproportionately impacted by GBV, with prevalence rates of up to 50% in some countries. In LMICs, however, the true extent of GBV is difficult to quantify due to several reasons, including lack of reliable data and the often-hidden nature of this form of violence [2, 16]. Tools and methods to capture the prevalence of GBV are often inadequate [17, 18]; furthermore, cultural, historical, and legal understandings of GBV vary across regions and culture, compounding the risk of under reporting or double counting [19, 20]. The under reporting of GBV is a critical challenge in LMICs due to cultural and social norms that discourage women from reporting abuse [3]. Women may face various barriers, including fear of stigma and shame, financial constraints, lack of services, fear of revenge, limited law enforcement action, and societal attitudes that normalise violence [4, 21].

Women and girls play a vital role in the economic, social, and cultural life of rural communities in LMICs. Rural women make significant contributions to household income through their participation in agricultural and other economic activities, such as home-based enterprises and small-scale agricultural ventures [3]. Globally, 36% of women work in agrifood systems; however, women are more likely to be employed in less-profitable value chains and activities due to traditional social norms or poor access to assets and resources [22]. In LMICs, in addition to agricultural roles, women are traditionally assigned to household work, child and elder care responsibilities, and other unpaid care work due to existing gendered divisions of labor.

Feminist economists refer to the concept of a “care economy” to describe the invisible and unpaid work undertaken by women globally [23]. While the valuable contributions of rural women are often overlooked and undervalued, their work is fundamental to the functioning of families, communities, and societies [24]. Rural women can also act as community leaders, change agents and decision makers, serving as role models for women and girls and breaking down barriers that hinder their full participation in society [25]. In LMICs, where rural women play a significant role in the social system and family economy, GBV limits their potential and has negative impacts on their well-being [16, 26]. Addressing GBV, therefore, is critical to creating safe and inclusive environments for women to participate fully in rural communities and contribute to economic, social, and cultural development.

While women’s contributions are central to the food and nutrition security of households and communities, the gendered nature of food systems is well established [16]. Women producers face unique challenges which limit their ability to fully participate in and benefit from agricultural activities [6]. Challenges include limited access to land, credit, and other resources necessary for agricultural production, as well as gender-based discrimination and limited participation in decision-making processes [5, 25]. Women producers frequently lack access to education and training opportunities, which can hinder their ability to adopt new technologies and practices to improve productivity and profitability [27]. Additionally, women may face discrimination and harassment in their work, as well as limited access to markets and other economic opportunities [28].

While there are some programs and strategies that have been implemented to address GBV in agricultural settings, such as microfinance programs, advocacy for equal land rights and the promotion of gender-sensitive training [25, 29], GBV remains an under-recognised reality, compounding the existing challenges faced by women producers in LMICs. As noted by Okpara and Anugwa, “GBV is not only a human rights violation, but also a catalyst to social degradation and food insecurity” [28] (p.12). The significance of addressing GBV, particularly in the context of women producers in LMICs, cannot be overstated. While research on GBV in agricultural settings is growing, it remains relatively underexplored [16, 30]. To make meaningful progress, more research is needed to understand the relationship between GBV and agriculture and gain insight into the impact of GBV on women producers in different regions and contexts. By undertaking a comprehensive review of the existing literature on GBV in the context of women primary producers in LMICs, this study aims to identify research gaps and priorities, and contribute to the advancement of knowledge in this field.

## Method

Scoping reviews serve as valuable tools in health research, enabling researchers to map essential concepts and identify gaps in the existing literature. As the impact of GBV on women producers remains poorly understood, a scoping review was deemed appropriate as it provides a comprehensive overview of the available literature, including its volume, nature, and characteristics, while also revealing areas where research gaps exist. To conduct the scoping review, the frameworks developed by Arksey and O’Malley [31] and Levac, Colquhoun, and O’Brien [32] were employed. These frameworks include, in broad terms, the following stages: [1] identification of research questions, [2] a search of the relevant databases, [3] selection of articles, [4] making a chart of findings from reading the articles and extracting relevant information, and [5] collection, summary, and report of the results.

### Research questions and study purpose

The scoping review process was guided by the following research question:

What is the current state of knowledge regarding GBV in the context of women primary producers in LMICs?

For the purposes of this scoping review, “women primary producers” were defined as women involved in agriculture and food production; this included plant production, livestock production, fisheries and aquaculture. In addition to this, the definition of food systems used by the Australian Centre for International Agricultural Research (ACIAR) and defined by the High-Level Panel of Experts on Food Security and Nutrition of the Committee on World Food Security (HLPE) was utilised [33, 34]. There was no threshold of involvement in primary production applied to inclusion; papers that discussed women involved in primary production whether as a full-time occupation or as one part of their livelihood activities, were included. If women had been involved in primary production but this involvement was curtailed because of GBV or GBV-associated factors, these studies were also included.

### Article search and selection

The project team consisted of three investigators with expertise on subject matter relevant to the scoping study. A librarian also assisted in performing the initial search and in refining search terms relevant to the research question. The search was conducted using PubMed, Web of Science and Ebscohost. Search terms relevant to gender-based violence, such as “Violence against Women”, “Domestic Violence”, “Intimate Partner Violence” and “Gender-Based Violence”, together with relevant acronyms were used. Search terms relevant to primary production, such as “farming”, “food production”. “agriculture”, “aquaculture”, “food systems” and “agribusiness” were also included. Only peer-reviewed journal articles published between January 2012 and June 2022 were included in the review. Through this initial search of three databases, 579 articles were found. Removal of duplicates using EndNote (a reference management software package) resulted in 452 remaining articles.

To be included in the review, papers had to meet the following inclusion criteria; (a) based on research in LMICs, as defined by the World Bank, (b) peer-reviewed journal articles, including systematic reviews, mixed methods, qualitative and quantitative papers, (c) published between 2012 and 2022, (d) considered food systems as per our definition, (e) considered GBV as per our definition, (f) and considered women and girls 15 and over (economically active) were included. Exclusion criteria included (a) men of any age, (b) grey literature, (c) research in High Income Countries (HIC), as defined by the World Bank and (d) articles not published in English. The team had initially planned to limit the scoping review to Melanesia but found only six suitable peer reviewed articles since 2012. The search was, therefore, expanded to all LMICs.

**Fig. 1:**
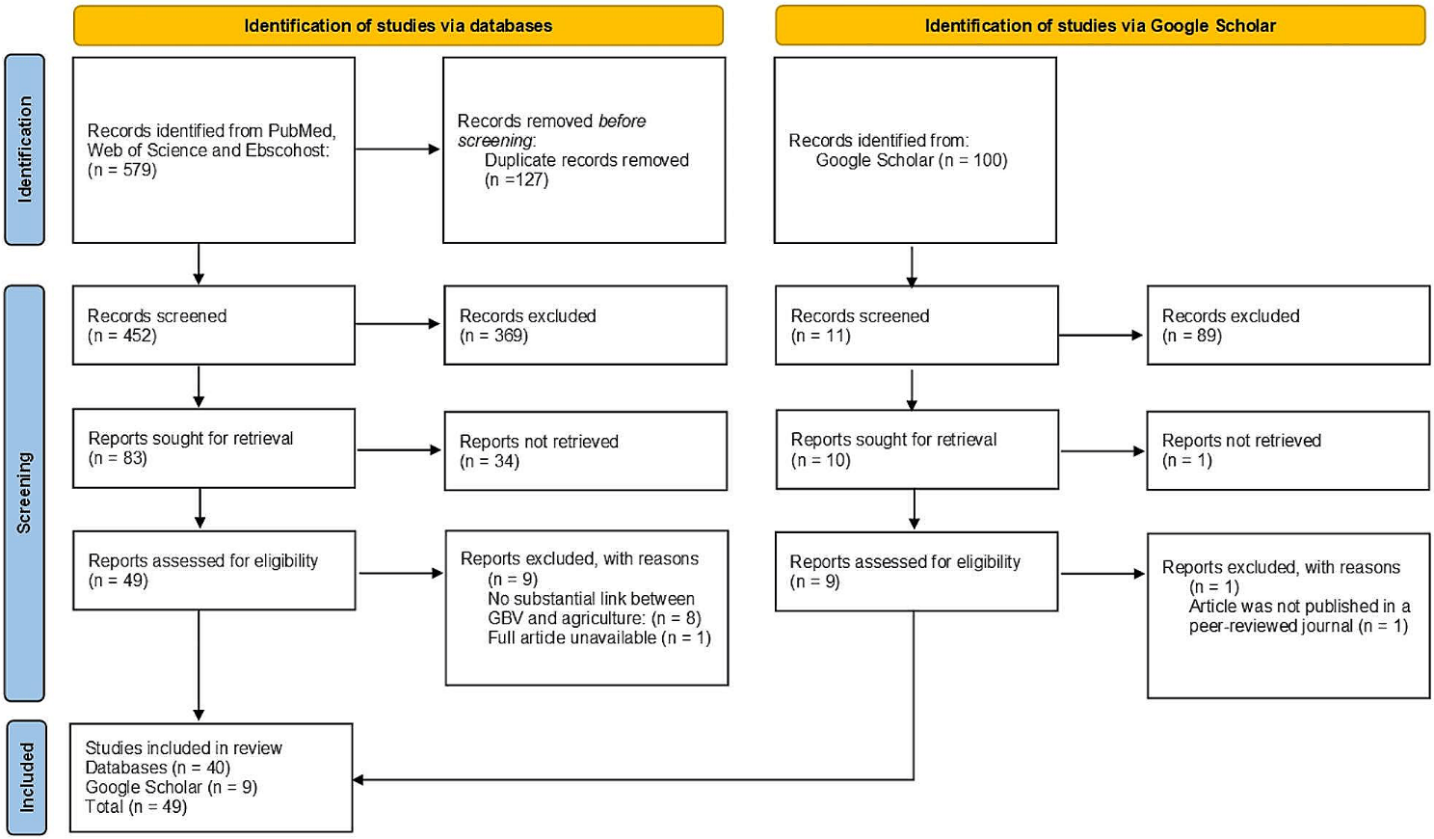
Article selection process conducted in this scoping review (adapted from the PRISMA flow diagram)

In the title and abstract round of reviews two investigators (CO & SS) independently reviewed paper titles and abstracts. This process yielded a total of 72 articles between both reviewers. Both investigators were in complete agreement on 35 of these articles for inclusion. Both investigators met to discuss discrepancies between their included/excluded articles. After considering the inclusion and exclusion criteria, together with the research question, both investigators reached a consensus on the inclusion of 49 papers. A Google Scholar search for any additional peer-reviewed journal articles not included in the initial database search was then completed using the terms: ‘“Gender Based Violence” “Women Farmers”. We included only the first 100 search results from Google Scholar for further rounds of title and abstract filtration. This process yielded a further 11 papers based on the inclusion criteria. One of these papers was already captured in the list of papers from the database searches and was therefore excluded. The combined 59 papers (49 from databases, 10 from Google Scholar) were then distributed among both investigators for a full text review, after which a total of 49 articles were deemed suitable for data extraction (See Fig. 1).

### Data extraction and analysis

The investigators collaborated to develop a data-charting form for tabulating relevant aspects of each reviewed article. In addition to author(s), year, title and journal, these data-chart categories were: country; aims of study; study population/sample size; methodology/methods; intervention type and duration (if applicable); outcomes; and key findings relating to the research question. Because of the volume of articles under review, two authors (CO and SS) divided the papers into two groups for data extraction. The authors met regularly to discuss progress and ensure that data extraction remained aligned with the research question (Levac et al., 2010). Data charts from both authors were combined and discussed jointly. During the data extraction process, broad themes were identified among the body of included studies. Five overarching themes (discussed below) were then identified; included articles were mapped against these themes. Many articles were assigned to more than one theme (See Table 1).

**Table 1:** Summary of studies

Citation	Country(s)	Aims of study	Study Population/Sample Size	Methodology/Methods/Intervention	Key findings that relate to scoping question.	Theme(s)
Adıbelli, D., Kırca, N., & Özkan, İ. (2022).	Turkey	To investigate the health and social challenges facing adult women working in greenhouse-based agricultural production in Turkey.	Women working in greenhouse agriculture over the age of 18.	Phenomenological study (qualitative) 22 participants.	Women engage in agricultural activities in addition to domestic duties. This double shift results in extreme physical fatigue and their reported lack of willingness to engage in sexual intercourse with their partners. Unwillingness to engage in sexual intercourse led to some men perpetrating violence against their wives.	Extent and nature.
Alesina, A., Brioschi, B., & La Ferrara, E. (2021).	Across Africa	To assess the relationship between cultural factors and current spousal violence in Africa.	DHS respondents in 21 Sub-Saharan Countries	Use of Demographic Health Survey (DHS) (which contains questions on domestic violence and attitudes towards domestic violence), matched with “Murdock’s Ethnographic Atlas”. Matching DHS and the ethnographic information created a pairing between ethnicity information with DHS responses.	Men have predominant role in the family and the research suggests that in these groups, DV is more common. The nature of agricultural work is also related to violence against women. Paper touches on the male backlash theory (economically independent women are seen as a threat to their partner)	Sociocultural; aggravating or protective factors.
Allen, E. M., Munala, L., & Henderson, J. R. (2021).	Kenya	To explore the relationship between severe weather events and intimate partner violence among Kenyan women.	DHS respondents in Kenya for 2008 and 2014.	DHS data collected in Kenya in 2008 and 2014 matched to GPS location of data collected for each DHS cluster. This allows for matching of DHS data about intimate partner violence (emotional, physical and sexual) with GPS locations, which in turn allow for matching with severe weather events.	Natural disasters and climate change exacerbate violence against women. Natural disasters in agricultural communities are correlated with IPV. The physical demands on women also increase with climate change.	Aggravating or protective factors.
Ashimolowo, O., & Otufale, G. (2012).	Nigeria	To assess domestic violence (DV) among women in Ogun State, Nigeria and explore socio-cultural factors and coping strategies.	220 women in farm families in Ogun State Agricultural Development zones. 30.80% of respondents were engaged in agriculture as an occupation.	Structured interviews.	To ameliorate the effect of DV on women’s agricultural livelihood activities, women adopt a range of coping mechanisms. These coping strategies impact agricultural livelihoods in different ways.	Impacts on livelihoods.
Austin, A., Schouten, C. N., Hinton, J., & Lloyd, D. J. (2021).	Papua New Guinea (PNG)	To investigate the barriers to inclusion in beekeeping faced by women in the the Eastern Highlands Province (EHP) of PNG.	Key industry stakeholders (*n*=23) in EHP. Focus Group discussions carried out with 9 participants from non-governmental organisations; workshops carried out with 12 female beekeepers.	Mixed methods. Semi-structured, key informant interviews. Focus group discussions with NGOs. Beekeeping workshop with female beekeepers.	Women are not given their fair share of income from beekeeping work. Men are more likely to support women engaging in beekeeping if they are included in the development of the process from the outset. Paper touches on important ideas of gender norms and ideas of “stopping women from participating” which could take on violent manifestations.	Impacts on livelihoods; aggravating or protective factors; sociocultural.
Ayuwat, D., & Sananikone, S. (2018).	Laos	To examine the factors underlying economic violence perpetrated by men against women in rural communities in Laos.	350 Lao men from households in the Santhong district of Loas.	Questionnaire	A high proportion of respondents were farmers. The paper highlights the nature of economic violence and control that some participants perpetrate against their wives, including control of their economic participation and the time they can spend outside, including engaging in farming activities.	Impacts on livelihoods; extent and nature.
Aziz, N., Nisar, Q. A., Koondhar, M. A., Meo, M. S., & Rong, K. (2020).	Pakistan	To investigate the relationship between women’s empowerment and food security in the rural areas of Azad Jammu & Kashmir.	600 households in 16 villages.	Household Food Insecurity Access Scale (HFIAS).	Paper draws a line of reasoning that women claiming land rights in highly patriarchal societies may be victims of domestic violence. There is a reluctance to claim land rights, which may impact food security and agriculture-related income, given the denial of productive land that would otherwise be claimed if there was not a concern of GBV.	Aggravating or protective factors; impacts on livelihoods.
Badstue, L., Petesch, P., Farnworth, C. R., Roeven, L., & Hailemariam, M. (2020).	Ethiopia	To examine the role of marital status in rural women’s innovative capacity in their agricultural livelihoods.	Women and men from various income and age backgrounds in eight community case studies in agricultural settings in Ethiopia.	Semi-structured life-story interviews; semi-structured innovation trajectory interviews; and focus group discussions to create case studies.	GBV plays a significant role in the lives of women respondents hampering their ability to engage in agricultural livelihoods, including through denial of land rights, physical violence, restrictions in mobility and sexual violence. GBV restricts women’s agency in agriculture and agricultural innovation.	Sociocultural; impacts on livelihoods; extent and nature.
Bonatti, M., Borba, J., Schlindwein, I., Rybak, C., & Sieber, S. (2019).	Tanzania	To explore the role of gender in food security among families in Dodoma, Tanzania.	Face to face survey of 333 households in two villages, Chinoje and Mzula, Tanzania. Followed by 2 workshops for 130 people.	Structured surveys, followed by workshops based on Theatre of the Oppressed (“Theatre of the Oppressed (TO). Workshops explored malnutrition, food behaviour and violence in context of food security.	GBV is normalised in some societies, especially with patriarchal social systems and hierarchies. Women cope with oppression and violence in several ways. There are also gendered and unequal roles in agriculture, such as men controlling cash crops and women controlling food crops. These and other arrangements are at times challenged, resulting in GBV.	Sociocultural; aggravating or protective factors.
Carter, N. A., Humphries, S., Grace, D., Ouma, E. A., & Dewey, C. E. (2017).	Uganda	To investigate the perceptions of smallholder pig farmers regarding the adoption of various innovations and changes related to a pig-enterprise and decision-making mechanisms and the role that gender plays in decision-making.	Focus groups with 72 villagers from Masaka district in Central Region, Uganda.	Intervention involved the delivery of lecture-style training to local pig producers, mostly on the topic of pig nutrition and feeding Participants arranged into three gender-stratified focus groups: men (*n*=24), women in male-headed households (*n*= 24) and women in female-headed households (*n*=24) to discuss perception of the potential impacts of adoption of improved diets for pigs and potential risks and risk mitigation associated with adoption of improved diets for pigs.	GBV plays a role in women’s decision making to buy pigs or engage in pig businesses (GBV is used by men to assert decision making status, and this extends to decisions on agricultural livelihoods). This is particularly pronounced in women in male headed households.	Extent and nature; impacts on livelihoods.
Carvalho, L. M. d., & Bógus, C. M. (2020).	Brazil	To explore and analyse women’s participation in a grassroots urban agroecological network in São Paulo, Brazil.	Attendees at workshops on women’s engagement in urban agriculture in Sao Paolo City, Brazil.	Participant observation at five women’s workshops and interviews with two organisers and three participants.	Women use spaces for training and discussion on agriculture to discuss sensitive issues. The presence of men at such spaces is met with resistance. This led to the formation of an “agroecological popular feminist identity”, freedom from oppressive social structures and a sense of collective belonging, centred on the women’s work in urban agriculture.	Extent and nature; aggravating or protective factors.
Chen, Y. J., & Chindarkar, N. (2017).	India	To investigate the impacts of a skills training program on the socioeconomic status of rural women in two sub-districts of Sabarkantha, Gujarat, India.	405 people from 37 villages, 212 women, 193 men (husbands of women or their adult son).	Survey	Training results in increased intra-household conflict, which, as other research has found is common when women find increased economic opportunity and/or entre the labour market.	Aggravating or protective factors.
Chipuriro, R. M., & Batisai, K. (2018).	South Africa and Zimbabwe	To describe the nature of the impacts of GBV on women related to land use in South Africa, Zimbabwe and elsewhere.	21 women farmers over 55 in Mashonaland Zimbabwe. AND six [6] elderly women farmers aged between 55 and 72 from KwaZulu-Natal, South Africa. Additional interviews with key informants.	Interviews, focus group discussions and life histories.	Patriarchal land control structures are associated with GBV. GBV is also perpetrated by male family members and neighbours with regards to issues of land access and land boundary disputes. Injuries from attacks, especially for elderly women, reduces productivity and full participation in agriculture, entrenching economic difficulties	Sociocultural; impacts on livelihoods; extent and nature.
Colfer, C. J. P., Achdiawan, R., Roshetko, J. M., Mulyoutami, E., Yuliani, E. L., Mulyana, A., Moeliono, M., Adnan, H., & Erni. (2015).	Indonesia	To understand the role that women play in decision making at various levels and to understand why women were active in decision making but invisible in other settings, such as formal meetings.	Thirty individuals from each of five villages in South Sulawesi and Southeast Sulawesi. Individuals were selected in a stratified random manner, 15 of each sex (from different households).	Survey (using a 0 to 10 scale) on people’s perceptions of their decision-making roles in four areas: food production and consumption; money management; life chances; and attitudes towards domestic violence.	The paper draws a link between decision-making and empowerment. While the paper indicates that GBV is highly unacceptable it also suggests that such unacceptability does not act as a proxy for equality in aspects of life and decision making. Paper also indicates that a strategy to work with men and women separately in carrying out agriculture-related programs may be a more effective strategy in settings where men hold unequal power.	Sociocultural; interventions.
Cooper, M., Sandler, A., Vitellozzi, S., Lee, Y., Seymour, G., Haile, B., & Azzari, C. (2021).	Global	To investigate the relationship between drought and the risk of intimate partner violence.	63 DHS surveys from sub-Saharan Africa, South and Southeast Asia, and Latin America and the Caribbean to assemble a data set of 363,428 women from 40 countries from the years 2000 until 2018	Demographic and Health Surveys (DHS) data was matched to Climate Hazards Group Infra-Red Precipitation with Stations (CHIRPS). Researchers used DV data from the DHS and drought-related data from CHIRPS. The authors ran logistic regressions pairing women’s DHS responses on experience with IPV (outcome variable) with CHIRPS data on drought conditions in the previous year (independent variable).	Drought is not related to emotional or physical violence (though this research is only in Africa, Americas, and Asia. Not in PNG or Pacific). The paper does describe how drought is associated with “controlling behaviours”, however, and this presents a need to explore how GBV is defined in different contexts.	Aggravating or protective factors.
Coulthard, S., White, C., Paranamana, N., Sandaruwan, K. P. G. L., Manimohan, R., & Maya, R. (2020).	Sri Lanka and India	To explore the link between alcoholism and domestic violence in fishing households in Sri Lanka and India	Women in fishing villages in Sri Lanka (*n* = 30) and India (*n* = 20)	In depth semi-structured interviews with women in Sri Lanka (near Rekewa Lagoon) and India (near the Gulf of Mannar Biosphere Reserve in Tamil Nadu), conducted over an eight-month period.	Women living with alcoholism and DV benefited from financial autonomy afforded to them through seaweed harvesting important. This activity allowed women to boost their status in the household and ensure their income is not spent on alcohol. Fisheries systems may be more vulnerable to high levels of alcoholism and domestic violence than others, hence, marine resource management should consider social well-being in fisheries.	Impacts on livelihoods; aggravating or protective factors.
Crookston, B. T., West, J. H., Davis, S. F., Hall, P. C., Seymour, G., & Gray, B. L. (2021).	Burkina Faso	To determine if the Building the Resilience of Vulnerable Communities in Burkina Faso (BRB), a project integrating financial services, women’s empowerment, nutrition and agricultural programs can improve women’s empowerment.	At baseline, the project included 760 participants, which reduced to 694 by the end of the study. Treatment group was drawn from the Sanguié province of Burkina Faso, and the comparison group members lived in the Nayala province of Burkina Faso.	Participants were divided into treatment and control groups. Treatment group received a comprehensive intervention package consisting of agriculture loans and services, microenterprise loans, and women’s empowerment programs. All groups completed the PRO-WEAII questionnaire at baseline (May 2016) and endline (November 2017).	Women were more likely to have adequate empowerment in input in productive decisions, group membership, and membership in influential groups than men while men were more likely to have adequate empowerment in attitudes about domestic violence, control over use of income, and work balance than women. BRB intervention may provide some protection for the treatment group when facing an economic down-turn.	Sociocultural.
Eneyew, A., & Mengistu, S. (2013).	Ethiopia	To assess the causes and extent of gender inequality through selected socio-economic factors in pastoral and agro-pastoral societies.	197 households, randomly selected from 3876 households, in South Omo, Ethiopia.	Interviews with households using the Harvard Gender Analysis Framework. In addition to this, key informant interviews and Focus Group Discussions were conducted in each village. Plus, a separate FGD for women, as the investigators identified that women were not permitted to speak in meetings if men were present.	Physical and psychological violence towards women is common in the study area, including female genital mutilation, wife beating and restricted access to education for girls.	Extent and nature; sociocultural.
Eves, R. (2021).	Papua New Guinea	To examine the marital relationships in Papua New Guinea’s Eastern Highlands and explore sexual violence within these marriages.	Qualitative interviews with 64 participants (coffee farmers), questionnaires with 143 households (including 124 married couples). 35 key informants drawn from the community, including village leaders, church leaders, women’s group leaders and village court officials.	Mixed methods - qualitative interviews and a questionnaire. The questionnaire contained three instruments “jointly administered to couples by a male and a female researcher, and separate male and female questionnaires administered by the male researcher and the female researcher respectively”. The female questionnaire asked additional questions relating to experiences of violence.	A quarter of both men and women responded that violence perpetrated by husbands on their wives is acceptable if the wife refuses sex. This informs understanding about attitudes and norms related to violence in sexual relations in coffee farmers in PNG. Bride price was paid in the case of 77.4% of couples; this is associated with attitudes about the rights that a man has over the body of his wife, including the husband’s right to beat his wife. The study discusses the role of land and that marriage forms the only pathway for a woman to access land and related resources - women are dependent on their husbands for survival.	Sociocultural; impacts on livelihoods; extent and nature.
Ezirigwe, J. (2018).	Nigeria	To examine (and challenge) practices that hinder participation of smallholder women farmers in Nigeria.		Literature review/essay	GBV limits women farmers’ productivity. There is weak enforcement of laws on DV, and law enforcement personnel promote the notion that DV should be dealt with within the family and not as a crime. Food scarcity and “lateness in preparing food due to time spent on collecting firewood “increase GBV risk. Safety risks while working on farms means women are forced to engage in agricultural tasks in the company of men, presenting a further challenge. Sparse medical services in rural areas means that medically establishing a rape or other crime is difficult; remoteness of the setting means a lack of witnesses.	Impacts on livelihoods; aggravating or protective factors.
Fischer-Daly, M (2022).	USA, Spain and **Mexico**	To analyse the role of dignity in bargaining power in the strawberry sector	154 workers, unionists, managers, government officials, scholars and activist in strawberry production.	Case study interviews.	Prevention of GBV perpetrated at work included as a key demand in collective bargaining of agricultural workers. Association between dignity and GBV. Sidelining of women in industrial action.	Interventions; impacts on livelihoods.
Handebo, S., Kassie, A., & Nigusie, A. (2021).	Ethiopia	To assess help-seeking behaviour of women who experienced physical and sexual violence	Data was extracted from the 2016 Ethiopian DHS dataset. The final analysis of the research was based on the responses of 1540 reproductive age women in Ethiopia who had experienced physical and sexual violence.	The study draws on the DHS data of the 1540 women (described in the Study Population/Sample Size column) to explore their help-seeking behaviours responses.	The likelihood of help seeking is higher in women working in agriculture compared to women who do not work. However, women whose *husbands* work in agriculture were 64% less likely to seek help than those with unemployed husbands. The authors state that women working in agriculture (and other sectors) might have higher help-seeking behaviour since they generate their own income and have more independence to seek help.	Sociocultural; aggravating or protective.
Hillenbrand, E., Lakzadeh, P., Sokhoin, L., Talukder, Z., Green, T., & McLean, J. (2014).	Cambodia	To employ Naila Kabeer’s Social Relations Approach (SRA) in implementing a baseline gender analysis of the Fish on Farms (FoF) project in Cambodia, and to assess the impact of the FoF project on women’s empowerment.	120 respondents. Two groups of 60 based on the two locations of the treatment arms of the FoF project randomised control trial. Participants included women involved in the project, their husbands and sometimes their grandmothers.	The Social Relations Approach (SRA) to conduct baseline gender analysis with approximately 120 participants in the two treatment arms of the FoF project randomised control trial. SRA uses interviews, group interviews and tools that encourage dialogue and involvement of respondents.	While there are some persistent aspects of GBV, participants recognized that women and men have equal rights. Unintended change outcomes of the FOF program can include women identifying a need for a reduction in GBV. Paper highlights need to provide spaces for all interview/discussion participants to voice their thoughts (potential to separate men and women for interviews).	Sociocultural; aggravating or protective factors.
Jalal, C. S., Frongillo, E. A., & Warren, A. M. (2015).	Bangladesh	To investigate how the Challenging the Frontiers of Poverty Reduction-Targeting the Ultra Poor (CFPR-TUP) program, a poverty alleviation program in ultra-poor Bangladesh, impacts two aspects of psychosocial health: distress and subjective well-being	Two hundred and nine women from three northern districts in Bangladesh: 110 women from the CFPR-TUP program households and 99 women in the control group (non-program households).	A baseline survey was conducted in 2002 with an endline survey in 2006. The survey included questions about the household, well-being, DV, food insecurity, distress, economic constraints, emotional constraints, and social constraints. One control group and one group engaged in program: Challenging the Frontiers of Poverty Reduction-Targeting the Ultra Poor (CFPR-TUP)	Poverty alleviation programs can reduce domestic violence, a key stressor for wellbeing. Engagement in the CFPR-TUP program led to a reduction in experiences of DV and food insecurity, highlighting options to implement interventions that address multiple aspects of women’s and households’ psychosocial health.	Interventions.
Jenderedjian, A., & Bellows, A. C. (2021).	Armenia & Georgia	This study draws on perspectives of non-governmental organizations (NGOs) in Armenia and Georgia to investigate NGOs’ *resistance to*, versus *embrace of* gender mainstreaming in rural development and agriculture.	215 surveys of NGO employees (food and nutrition focused); 53 interviews with NGO employees (24 Armenian; 29 Georgian)	Applying Bourdieu’s theory of field-habitus, and intersectionality as critical praxis. Uses sequential mixed methods (Electronic survey and individual interviews)	Gender mainstreaming (GM) is important within food security programs but not widely accepted. There is a need to raise awareness of the importance and value of GM before we start discussing “how to do” gender or equality mainstreaming. Feminist NGOs recognise that GBV is considered a necessity rather than an ethical dilemma or unwelcome move. This is in stark contrast to resistance expressed by male leaders of non-feminist NGOs.	Interventions.
Kohli, et al. (2015).	Democratic Republic of the Congo (DRC)	This study explores risk factors, individual and family consequences and community-driven responses to IPV in post-conflict eastern DRC.	Participants included 13 female survivors and 5 male perpetrators of IPV as reported during baseline data collection as part of the “Pigs for Peace” livestock microfinance program	Qualitative: in-depth interviews with rural men and women involved in the Pigs for Peace village-based microfinance program.	Culturally acceptable, community-based prevention and response programs to combat IPV are recommended. Interventions should be developed with the community in a participatory manner.	Aggravating or protective factors; interventions.
Kumar, N., Raghunathan, K., Arrieta, A., Jilani, A., & Pandey, S. (2021).	India	To assess the impact of women’s membership in a self-help group (SHG) on women’s empowerment.	Panel data on 1470 rural Indian women, 1344 rural Indian men	Baseline survey: household socioeconomic and demographic characteristics, participation in SHG platforms and women’s empowerment. Use of Women’s Empowerment in Agriculture Index	SHG membership does not appear to significantly impact attitudes towards IPV. However, there is some evidence that IPV can be reduced through cash and in-kind transfers to women. However, such impacts are short-lived and not sustained beyond the life of a project. It is Important for measurement of IPV to use adjusted tools (e.g. WEAI) for project impact assessment.	Interventions; extent and nature.
Maduekwe, E., et al. (2020).	Malawi	To illustrate the extent of women’s human recognition in Malawi and to contribute to the body of knowledge on measuring human development for policy designs.	Malawi’s DHS data: 2004, 2010, 2015	Use of the Alkire-Foster method (for measuring poverty or wellbeing) to estimate human recognition deprivation index (HRDI) among women in Malawi. The DHS includes questions regarding a respondent’s experience or perception of several domains related to human recognition, including those dealing with violence, humiliation, dehumanization, and autonomy.	Women working in agricultural settings are valued less by society.	Sociocultural.
Mahmud, M., & Riley, E. (2021).	Uganda	To explore the economic and well-being impact of the COVID-19 lockdown on a sample of households in rural Uganda.	Initial household survey of 1277 households prior to lockdown (March 2020). Further phone survey after lockdown with 85% sample (May 2020)	Face-to-face (F2F) survey and follow up phone survey.	Economic security and emotional well-being are key pathways impacting on violence. Women faced worsened conditions in the home and are at an increased risk of violence resulting from extreme shocks and reductions in farm income. Targeted income and food consumption support may help mitigate some of these impacts.	Aggravating and protective factors; interventions.
Malapit, H., Ragasa, C., Martinez, E. M., Rubin, D., Seymour, G., & Quisumbing, A. (2020).	The Philippines	To adapt the survey-based project-level Women’s Empowerment in Agriculture Index (pro-WEAI) to measure women’s and men’s empowerment in value chains and to investigate correlates of empowerment.	1264 households and 2811 individuals were interviewed for the survey. 40 qualitative interviews were undertaken.	Pro-WEAI, a survey-based tool to measure women’s and men’s empowerment and inclusion in agricultural development projects was adapted to focus on aspects of empowerment relevant to value chains (VC). Survey design was informed by initial qualitative interviews. Qualitative interviews were also conducted after survey to provide insights into some of the key results and patterns emerging from the pro-WEAI scores.	Within VC context, respect within the household and attitudes about GBV are the largest sources of disempowerment for both women and men, followed by control over use of income and autonomy in income-related decisions. Gender issues exist across VCs, highlighting the need to address deeply rooted, structural gender and social norms.	Sociocultural; extent and nature
Meinzen-Dick, R., Quisumbing, A., Doss, C., & Theis, S. (2019).	Global	To review literature on women’s land rights (WLR) and poverty reduction to identify pathways by which WLR could reduce poverty and increase wellbeing of women and their households in rural areas.	52 references used for review	A systematic review to assess the available high-quality evidence on the effects of strengthening WLR on development outcomes related to poverty reduction	The review includes papers that examine the relationship between WLR and domestic violence, one of the clearest indicators of disempowerment. Three papers in the review show significant links between land ownership, relationship power and GBV. Land ownership is negatively associated with physical and psychological violence.	Sociocultural; impacts on livelihoods; protective
Narang, B. (2014).	India	The study aims to explore the effect of water scarcity on the perceived health status and quality of life of people in a rural village in Mewat.	Numbers not outlined in paper	This study is based on an inductive approach, focus group discussions, participatory exercises, and dialogue with key informants as primary modes of data collection.	Collecting water from outside the village took a toll on women and impacted on productive time. Water scarcity led to increased stress, including increases in GBV.	Aggravating or protective factors.
Nerkar, S. S., Tamhankar, A. J., Johansson, E., & Stålsby Lundborg, C. (2013).	India	To understand perceptions of the tribal community members regarding public health and socioeconomic implications of watershed management (watershed = in an area or a region which contributes rainfall water to a river or lake.)	Six focus groups	Qualitative study with six focus group discussions (FGDs), three each separately for men and women. Conducted among tribal community members of the Maharashtra state of India.	Watershed management could directly or indirectly result in reduction of the public health related challenges in this setting, such as alcoholism, intimate partner violence, as well as drudgery of women. There are links to climate vulnerability with environmental stressors impacting on GBV.	Interventions.
Otufale, G. (2013).	Nigeria	To analyse socio-cultural factors influencing GBV on agricultural livelihood activities of rural households in Ogun State Nigeria.	220 rural women surveyed from 3 zones	Quantitative study using multistage sampling. Women involved in agricultural livelihood activities.	GBV negatively impacts on agricultural livelihood activities. Societal and cultural norms impact on GBV highlighting the need for programs to identify, understand and address sociocultural factors	Impacts on livelihoods.
Quisumbing, A., Ahmed, A., Hoddinott, J., Pereira, A., & Shalini, R. (2021).	Bangladesh	To assess the impacts of agriculture, nutrition, and gender interventions on women’s empowerment in Bangladesh.	25 households involved in training. Empowerment measured through pro-WEAI tool.	A clustered randomized controlled trial with the following arms was conducted July 2016 to December 2017. T-A: Agricultural Production training T-N: Nutrition Behaviour Change Communication (BCC) T-AN: Agricultural Production training and Nutrition BCC T-ANG: Agricultural Production training, Nutrition BCC, and Gender Sensitization C: Control	Interventions improved women’s empowerment but findings on whether GBV is reduced are not straightforward. Paper highlights importance of monitoring unintended harm to women when implementing gender-transformative interventions. The role of engaging men and women jointly in interventions, and how best to do so, is a promising area for future research.	Interventions.
Ragetlie, R., Hounkpatin, W. A., & Luginaah, I. (2021).	Sub Saharan Africa (Benin)	To explore the connection between alcohol misuse and food insecurity in the Atacora region of Benin.	20 interviews with couples from farming families [35] pus 6 community focus groups	Qualitative study (40 participants for dyad interview; 94 participants in focus groups)	Highlights the complex interplay between food insecurity and GBV. In lean times, heavy drinking is problematic, sparks arguments leading to increasing GBV. More women using alcohol to cope with stress and hunger; therefore, alcohol misuse is impacting women farmers too. Alcohol misuse needs to be addressed as part of a broader strategy to address GBV in the context of rural livelihoods.	Aggravating or protective factors.
Ragetlie, R., Sano, Y., Amoussa Hounkpatin, W., & Luginaah, I. (2021).	Benin	The study aims to investigate the association between household food production and IPV in Atacora, Benin.	300 rural women surveyed	Quantitative: Using a social ecological model and drawing from family stress theory, data from a cross-sectional survey of 300 women in the study region collected and analysed.	IPV is more of a widespread problem among farmers than population-level reporting indicates. Study points to the importance of addressing women’s access to, and ownership over, land as a strategy to address IPV. Involving more women in production-oriented interventions in rural farming communities may reduce women’s risk of IPV by increasing households’ access to food and reducing family stress.	Extent and nature; interventions; aggravating or protective factors.
Rignall, K. E. (2019).	Morocco	An essay which explores the analytical consequence of considering structural violence of rural life through a gendered intersectional lens.	Ethnographic research	Fieldwork/observation	This research on structural violence indicates the extent to which women‘s participation in agricultural production and other economic activities is rendered invisible by formal statistical categories. Most women in the valley participate in agricultural production but that participation may be invisible in data collected. Need better data capturing approaches.	Extent and nature.
Sabri, B., Wirtz, (A) L., Ssekasanvu, J., Nonyane, (B) A. S., Nalugoda, F., Kagaayi, J., Ssekubugu, R., & Wagman, J. A. (2019).	Uganda	This study aims to identify unique correlates of IPV, HIV and sexually transmitted infections (STI) in fishing, trading and agrarian communities in Rakai, Uganda	Survey of 14,464 individuals	Quantitative study - cross sectional data collected from a survey of 14,464 men and women from fishing, trading and agrarian communities in Uganda.	Social norms play an important role in IPV victimization and justification. Justification of IPV appears to increase risk for IPV perpetration among men in the fishing community. Social norms that allow and condone IPV must be considered in developing appropriate strategies. Alcohol use can also place individuals at risk of IPV - programs need to target multiple factors and not address IPV in isolation.	Sociocultural; aggravating or protective factors.
Sadati, S. M. H., & Mitchell, C. (2021).	Ethiopia	Ethiopia has one of the highest rates of sexual and gender-based violence (SGBV) in the world. Study explores how instructors in agricultural colleges understand GBV and its causes and explores the role of instructors as ‘agents of change’.	Interviews with 20 participants, and other narrative based methods	Qualitative: Narrative based methods including interviews and an interactive storyline development workshop, as well as cellphilming (cellphone + film) as a participatory visual method.	Agricultural colleges can play a key role in delivering GBV interventions. They can be places for learning and spaces of transformation.	Interventions.
Sadati, S. M. H., & Mitchell, C. (2021).	Ethiopia	Ethiopia has one of the highest rates of sexual and gender-based violence (SGBV) in the world. This study explores the design of a game called *Mela* through ‘research-based creation’ and the use of arts-based practices.	N/A	Explores the research process and steps involved in creating a game to help ag college instructors address issues relating to GBV.	Mela as a serious game has been designed to address GBV in Ethiopian agriculture colleges. The idea of game development in a LMIC is more male at least in terms of prevailing gender norms and may offer a creative approach to addressing GBV in agricultural settings.	Interventions.
Sandberg, J. F., Delaunay, V., Boujija, Y., Douillot, L., Bignami, S., Rytina, S., & Sokhna, C. (2021).	Senegal	To test a series of models of social learning related to the acceptability of spousal IPV using survey data from a population in rural Senegal.	Three linked sources of data. In total, the analytical sample size is 1274 individuals residing in 193 compounds across 10 neighbourhoods.	Uses linked data from NSNHP survey data, NDHSS surveillance data and a census of household wealth.	Those whose household wealth was more concentrated in agricultural production were more likely to hold more traditional gender schemas and be supportive of IPV than those whose households were less so. Social norms are important and need to be considered when trying to change attitudes about GBV. Interventions targeting individuals (*both men and women*) who are likely to perceive GBV as acceptable may have a multiplier effect.	Aggravating or protective factors; sociocultural; interventions.
Simmons-Stern, L. (2013).	Cote d’Ivoire	Study aims to evaluate the incremental impact on levels of IPV of adding “Gender Dialogue Groups” for women and their partners (aiming to change gender norms) to an economic empowerment program for women in rural Cote d’Ivoire.	Thirty rural villages. Intervention included 981 rural women in first intervention (513 treatment; 421 control) 934 women (95.2%) completed baseline and follow up survey/data collection	A two-armed, non-blinded randomized-controlled trial (RCT) comparing group savings only (control) to “gender dialogue groups” added to group savings (treatment). The gender dialogue group consisted of eight sessions that targeted women and their male partner. Eligible Ivorian women (18+ years, no prior experience with group savings) were invited to participate.	Findings illustrate the potential benefits of *adding gender sensitization components into livelihood programs* for women in conflict and non-conflict affected settings. While economic empowerment programs targeting women are advancing women’s status, health, and livelihoods, the addition of an intervention for women and their male partners to promote gender equitable norms yields more reductions in IPV than economic programming alone.	Interventions.
Simsek, Z., Kara, B., Ersin, F., Okten, S., & Yildirimkaya, G. (2016).	Turkey	Study determines male and female seasonal agricultural workers’ perception of violence, prevalence of violence, frequency of victimization, and their related factors	2300 surveys (male and female), two focus groups	Mixed methods - quantitative (survey) followed by two focus groups with women impacted by GBV.	Economic violence is particularly high. This is possibly associated with the fact that working women mostly have no say in how their earnings are used. A significant number of women approve of or tolerate violence, highlighting the importance of education and raising awareness. Education is a protective factor against GBV - key strategy to consider.	Extent and nature; sociocultural; aggravating or protective factors.
Vedhanayagi, P. (2013).	South India	Study outlines the development and early success of the Thendral movement which encompasses people-centred activities to empower women and nurture their role in agriculture.	N/A	Autoethnography detailing the author’s experience of developing an agro-feminism movement “Thendral movement’ in South India	Women’s movements and solidarity between women may be an important step for starting to address specific problems that women in agriculture face.	Interventions.
Waid, J. L., Wendt, A. S., Sinharoy, S. S., Kader, A., & Gabrysch, S. (2022).	Bangladesh	The aim is to evaluate the impact of a 3-year homestead food production program on men and women’s agency. FAARM program	pro- WEAI data (women’s empowerment in agriculture tool) collected from 885 participants.	Cluster-randomized controlled trial with intervention (FAARM program) to support gardening and poultry rearing. A three-year intervention Food and Agricultural Approaches to Reducing Malnutrition (FAARM program) with groups of 16 women.	Programs focusing on developing women’s agency may have a positive impact on attitudes towards GBV and may reduce some of the risk factors for GBV (i.e., by increasing equity between spouses and increasing self-efficacy).	Interventions.
Wilson, J. B., Rappleyea, D. L., Hodgson, J. L., Hall, T. L., & White, M. B. (2014).	N/A	The aim is to review previous research related to IPV screening among Migrant and Seasonal Farm Worker (MSFW) women and recommend useful policies.	Literature search - five studies	Literature review	IPV rates are higher in this population, but women farm workers lack support and resources. There appear to be limited screening tools to detect IPV, so prevalence is typically under reported.	Extent and nature.
Yount, K. M., Cheong, Y. F., Maxwell, L., Heckert, J., Martinez, E. M., & Seymour, G. (2019).	Bangladesh & West Africa	To obtain baseline data from two studies to assess pro WEAI measurement tool. Studies were (i) Targeting and Realigning Agriculture for Improved Nutrition (TRAIN) in Bangladesh and (ii) Building Resilience in Burkina Faso (BRB).	TRAIN survey was administered to 5040 households in which at least one woman 18–35 years was present. BRB survey was administered to a subset of households, including 380 women (190 intervention group; 190 comparison group), as well as their husbands or the male heads of household (a total of 760 respondents). Only the women’s responses were analysed in this study.	Quantitative: baseline data from two GAAP2 projects analysed to assess measurement properties in pro-WEAI.	The IPV attitude item " capturing intrinsic agency in the right to bodily integrity” can be used unidimensionally but may not assess change over time. When considering pro-WEAI tool for assessing IPV attitudes, response items could be expanded.	Extent and nature.
Zafar, S., Saima Zia, M. S., & Amir-ud-Din, R. (2021).	Nineteen Developing Countries (South Asia, Sub Saharan Africa and Middle East)	This study analyses if women’s employment is significantly associated with their experience of IPV. Job status is disaggregated into three different categories: (i) agricultural jobs (AJ), (ii) blue-collar jobs (BJ), and (iii) white-collar jobs (WJ)	Data used for this study is taken from the Integrated Public Use Microdata Series of Demographic and Health Surveys (IPUMS-DHS). The study participants were ever-married women aged 15-49 years. Over two million women in 19 countries from 2010 to 2016 gave complete interviews (*N* = 2,237, 919).	The countries and corresponding years in which surveys were conducted include Burkina Faso (2010), Cameroon (2011), Congo, DR (2007, 2013), Cote d’Ivoire (2011), Egypt (2005, 2014), Ethiopia (2016), Ghana (2008), India (2005, 2015), Kenya (2003, 2008, 2014), Malawi (2004, 2010, 2016), Mali (2006, 2012), Mozambique (2011), Nigeria (2008, 2013), Pakistan (2012), Rwanda (2005, 2010, 2014), Tanzania (2010, 2015), Uganda (2006, 2011), Zambia (2007, 2013), and Zimbabwe (2005, 2010, 2015).	Women’s employment does not protect them against all forms of IPV. The relationship between women’s employment and their experience of IPV is context specific. Educating practitioners and policymakers to understand some of the nuanced IPV risks faced by working women may significantly decrease IPV risk.	Aggravating or protective factors; interventions.

## Findings/results

### Description of studies: populations, research designs

While the included 49 studies varied in their aims, all considered GBV within agricultural or rural settings. All studies that drew on primary data sought contributions from the economically active workforce (women or girls over 15 years). Often these were women primary producers, as defined above, or the study identified a subpopulation of women primary producers within the broader sample population. At times, male partners or other male household members were also included in the study. Other relevant stakeholders, such as educators, were at times included. The studies included in our scoping review employed a range of methods. While many were based on mixed methods, commonly interviews together with surveys and/or focus groups, studies utilizing only interviews or surveys/questionnaires were also common. Six studies drew on secondary data, particularly data from the Demographic Health Survey (DHS) [36]. Literature reviews, standalone focus groups, and observation-based studies were also included but not to the extent of other research approaches.

**Fig. 2:**
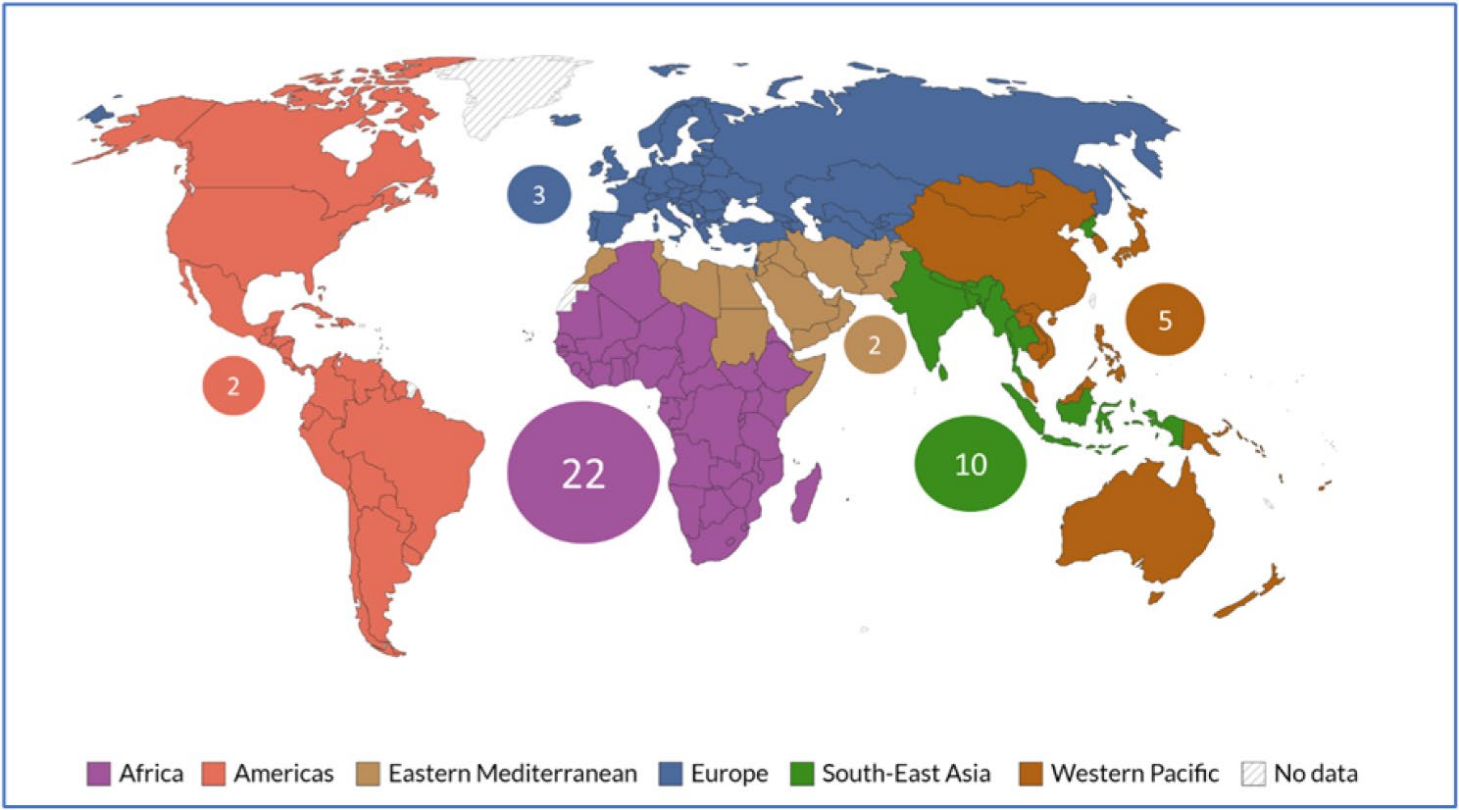
Number of peer-reviewed journal articles selected from each WHO region

Using the World Health Organization (WHO) six regional groupings [37], geographical classifications were assigned to the articles in this scoping review. These groupings are the Africa Region; Region of the Americas; South-East Asia Region; European Region; Eastern Mediterranean Region; and Western Pacific Region. While forty-four of the included papers could be placed in one of these regions, five articles, all literature reviews or studies involving secondary data, were based in more than one country and across two or more of these groupings. Twenty-two papers (44.9%) were based on research in the Africa Region, two articles (4.1%) were based on research conducted in the Region of the Americas; 10 articles (20.4%) in the South-East Asia Region; three (6.1%) in the European Region; two (4.1%) in the Eastern Mediterranean; and five (10.2%) in the Western Pacific Region. The African Region was by far the most represented across the articles in the scoping study (see Fig. 2).

The 5 major themes identified from our analysis are discussed below: [1] extent and nature of GBV, [2] the impact of GBV on agricultural/primary production livelihood activities, [3] sociocultural beliefs, practices, and attitudes, [4] aggravating or protective factors, and [5] GBV interventions. These themes are explained using a narrative approach.

### Extent and nature of GBV

The first theme, extent and nature of GBV, was assigned to papers that explored or highlighted the prevalence of GBV and/or the extent to which data on GBV is collected, and manifestations of GBV in the settings concerned. Papers that explored under-reporting or inadequate tools for capturing data were also included here. Our review categorized fifteen (31.25%) articles under this theme. In terms of extent, the literature revealed that GBV is prevalent in many primary production-based communities in LMICs, with numerous women reporting they had experienced or are currently experiencing GBV [38, 39]. However, due to the limitations in adequately capturing data on GBV in these settings, the true extent of GBV is likely underestimated. Of note, some papers discussing GBV in primary production settings in LMICs, specifically highlighted how poor screening methods or research approaches inadequately captured or ignored women’s experiences, therefore affecting the reliability of data [35, 40–42]. Under reporting of GBV is a critical challenge; several papers explored the use and efficacy of methods to capture data on GBV, including through surveys, interviews, focus group discussions and tools such as the Women’s Empowerment in Agriculture Index (WEAI) [11, 18, 38, 42]. These studies also explored inadequacies in fully capturing the true extent of GBV, indicating the actual prevalence is underestimated.

The nature of GBV encompassed various forms in the context of LMIC-primary production settings and included physical violence [38, 43–45] sexual violence [38, 43, 44], economic violence [44, 46–48] and psychological violence [38, 45]. In the realm of GBV reporting and monitoring, however, certain types of violence, such as physical violence and sexual violence received more attention and focus compared to other forms of violence. Psychological violence, for example, can be challenging to recognise, monitor and report and therefore hindered our understanding of the full scope of GBV in this setting.

Men perpetrate violence against women for various reasons, influenced by a combination of individual, social, economic and cultural factors. Our review highlighted some common underlying factors including refusal of partners to engage in sexual intercourse, to achieve a self-image of masculinity, and transgression of cultural norms related to women’s roles in society and primary production-related work [43–45]. In the study conducted by Simsek et al. [46] on seasonal agricultural workers in Turkey, husbands and/or fathers denied women the ability to make decisions about their earnings, which was identified as a form of economic violence. Similarly, in Papua New Guinea, Eves [44] highlighted the prevention of economic decision-making as a feature of GBV. This form of economic violence frequently affected women’s participation in agricultural livelihoods [48].

### The impact of GBV on agricultural/primary production livelihood activities

The second theme, the impact of GBV on agricultural/primary production livelihood activities focused on the impact of GBV on women’s engagement and participation. Within the scoping review, thirteen (27.08%) articles were identified. These articles revealed that GBV negatively impacted on women’s benefits from and engagement with agricultural and other primary production livelihood activities [49]. Furthermore, the articles discussed how GBV, and the associated power dynamics restricted women’s decision-making abilities, limited their access to resources, impeded their skills development, and hindered their agency. Within this context, several interrelated themes emerged, highlighting the complex way in which GBV affects women primary producers. One recurring theme was the physical and mental toll of GBV on women’s health, directly impacting their ability to work and engage in agricultural activities. Instances of morbidity resulting from GBV created barriers that limited women’s productivity and hampered their active participation in the agricultural sector [26].

Furthermore, women producers working in isolated settings faced an increased risk of sexual violence and other forms of GBV [26, 40]. The inherent vulnerability of their surroundings exposed them to heightened dangers, undermining their sense of safety and well-being [40, 45, 50]. Not surprisingly, the demands and pressures associated with primary production and household responsibilities, including food scarcity, exacerbated GBV. Women producers facing economic and emotional strain, were more vulnerable to GBV, further undermining their well-being, and hindering their productive contributions [26, 40]. In the context of GBV in agricultural settings, these interlinkages were particularly evident and created a vicious cycle that exacerbated the negative effects of violence on women producers.

Land rights, as a cultural phenomenon and a barrier, played a significant role in limiting women’s engagement in livelihood activities [25, 40, 51]. While land rights may not be inherently classified as GBV, the denial or restriction of women’s land rights can contribute to and perpetuate GBV. Cultural norms, traditions, and patriarchal systems often intersected with land ownership and inheritance, creating obstacles for women’s access to and control over land resources [40, 44, 51]. The theme of land rights and land ownership featured prominently in the literature and will be discussed again later in this paper.

Economic control, a recognised and insidious form of GBV, was also a recurring theme in the literature. Lack of economic agency, for example, withholding women’s farm income restricted their decision-making abilities, curtailed their economic mobility, and had the potential to stifle entrepreneurial and innovative potential [40, 44, 45, 47, 52]. Additionally, women faced limitations on the type of livelihood activities they could engage in due to gender norms and expectations; such limitations further perpetuated gender inequalities [47, 48, 52, 53]. Male producers, for example, were typically given control over cash crops or high-value agricultural activities; in contrast, women were often assigned tasks related to subsistence farming or low-value crops [47, 52]. This gendered division of labour reinforced gender disparities in agricultural productivity.

Coping strategies used by women to mitigate these impacts on agricultural livelihoods were also discussed. These strategies included fighting back against GBV and asserting rights, appeasing the perpetrator to minimise the occurrence of GBV and seeking social support from family and friends [54]. In some instances, women producers sought the intervention of law enforcement, resorting to police intervention, seeking counsel, and instituting legal action [53, 54]. Several articles highlighted strategies to mitigate the impact of GBV on livelihoods; these articles will be discussed later in this paper under the theme GBV interventions.

### Sociocultural beliefs, practices, and attitudes

The issue of GBV is multifaceted, and the third theme, sociocultural beliefs, practices, and attitudes, provided valuable insights into the underlying factors that contribute to GBV. Nineteen articles (39.6%) grouped under this theme highlighted how gender inequality underpinned GBV through factors such as culturally sustained gender norms and schemas, historical factors, traditional inheritance practices, policy and customary norms.

Research revealed that GBV is sometimes approved or tolerated by both women and men in certain contexts. Several studies, including those by Eves [44], Simsek et al. [46], Sandberg et al. [55], and Crookston et al. [56], highlighted this acceptance of GBV. Studies also highlighted the acceptance of GBV in response to specific behaviors, such as the refusal of sex [44]. Not surprisingly, sociocultural beliefs, practices, and attitudes were identified as influential in shaping the occurrence and acceptability of GBV. For example, households where wealth is derived from agricultural production were found to be more likely to exhibit attitudes that sustained GBV [55].

As highlighted by Maduekwe et al. [57], in research from Malawi, women who work in agriculture, are more likely to be “human recognition deprived’; that is, undervalued and under-recognised by society. Cultural norms and attitudes, in this context, were seen to support male dominance, foster conservative perspectives on women’s societal value, and potentially facilitate a more accepting environment for GBV. While one study from South Sulawesi reported strong disapproval of GBV, other studies highlighted the influence of social norms and the networks through which they were disseminated [11, 38, 55, 58–60]. These social norms created conditions that enabled GBV to persist not only in primary-producer settings but also throughout agricultural value chains.

The connection between agricultural livelihoods, history and tradition contributed to attitudes that sustained gender inequality and GBV [38, 59, 61]. Research demonstrates that in certain contexts, sociocultural factors shaped gender roles centered around responsibilities related to primary production [49, 59, 62]. These gender roles reinforced power inequalities, and further perpetuated GBV [38, 52, 60, 61, 63]. Of note, women’s engagement in primary production activities was, at times, perceived by men as a threat to their traditional masculine identities [53, 59]. A particularly notable association between sociocultural norms related to agriculture and GBV was observed by Alesina et al. [59]. This research, utilising the Demographic Health Survey [36] and Murdock’s Ethnographic Atlas [64] linked present-day GBV occurrences to historical forms of agriculture in Africa. Societies with historical forms of production such as plough agriculture, fishing, and husbandry (primarily male dominated) were found to have higher rates of GBV, even when present-day descendants were no longer engaged in primary production. Conversely, societies in Africa with non-plough-based agriculture, where agricultural tasks were historically more equally shared between women and men, exhibited lower rates of GBV [59].

Research revealed that land ownership is connected to agency and a woman’s economic independence; several articles explored the connection between traditional modes of land rights or land inheritance/ownership and GBV [25, 40, 44]. The association between traditional modes of inheritance or land rights and GBV, revealed two distinct outcomes for women producers. In cases where cultural practices provide women with land, these traditional practices contributed to increased agency and decision-making power for women [25]. Conversely, when women were excluded from inheriting land, this limited autonomy and had a detrimental effect on women producers. The exclusion of women from inheriting land may lead to a dependency on marriage as the primary means of acquiring a livelihood [40, 44] (Chipuriro, 2018; Eves, 2021). Of note, Mienzen-Dick et al. [25] (p77) described the relationship between women’s land rights and GBV as “one of the clearest indicators of disempowerment”. In their review of the literature on women’s land rights and poverty reduction, Mienzen-Dick and colleagues [25] revealed that women’s property (land or house) ownership is “significantly and negatively associated with both long-term and current physical and psychological violence.” In summary, the literature suggested that land ownership has the potential to protect against GBV.

The topics of bride price and marital customs also emerged within the literature. Three papers examined how these customs intersect with GBV in such communities [40, 44, 59]. Bride price, sometimes paid through the provision of livestock and agricultural goods, was associated with a sense of ‘ownership’ by men over their wives, including control over their bodies, time, labour and assets [40, 44, 59]. Chipuriro [40] described one case from Zimbabwe in which the husband of a woman who had harvested crops together with her children, took her harvest to the market himself and withheld the money from its sale from his wife. He then used this money to purchase cattle for “lobola” (payment in cattle or cash to a bride’s family shortly before the marriage) for new wives. Customs such as bride price reinforced attitudes regarding women as the ‘property’ of their husbands; furthermore, the requirement to repay bride price created further barriers for women seeking to leave abusive relationships [59].

### Aggravating or protective factors

The fourth theme, “aggravating or protective factors,” focused on examining individual and environmental factors that either aggravated or mitigated GBV. Unlike the third theme, which explored social and cultural norms, this theme delved into additional aspects that influence GBV. In this context, aggravating factors referred to any factor or condition that exacerbated or worsened the severity, frequency, or impact of GBV. Protective factors referred to any factor or condition that reduced the risk or impact of GBV. These protective factors differed from prevention strategies as they were not interventions designed to reduce the incidence or prevalence of GBV. Twenty-one papers (43.75%) were grouped under this theme.

We identified several aggravating factors that exacerbated the risk of GBV. One notable aggravating factor related to the historical forms of agriculture practiced in certain societies and discussed earlier in this paper [59]. In African societies, where plough agriculture, fishing, and husbandry have traditionally held prominence, there was a strong association between masculinity and these occupations. The pressure to conform to these masculine ideals may have contributed to the aggravation of GBV in this context [59]. Work in the agricultural sector, as opposed to the non-agricultural sector, was also identified as an aggravating factor. The challenging and hazardous nature of agricultural work, coupled with unequal gender roles, increased the vulnerability of women producers in such settings [55, 62]. In the context of primary production, alcohol abuse by intimate partners was a common aggravating factor in GBV situations. Several studies discussed how high levels of alcohol consumption, primarily among men, contributed to an environment of coercion, control, and physical harm [53, 58, 60, 61, 65–67]; all primarily among men authors advocated for interventions to address substance misuse.

Not surprisingly, challenging traditional gender roles and norms was recognised as an aggravating factor for GBV. Women who challenged gender roles or asserted their rights to land, for example, were more likely to trigger a backlash and increase the risk of GBV. Even small acts of defiance by women producers threatened existing power relationships and led to heightened tensions and escalating violence [53, 60, 67, 68]. Weak law enforcement and inadequate responses further aggravated GBV in such settings [26]. Concerningly, women who did report GBV, faced significant barriers to justice [26]. Other aggravating factors included household economic instability, extreme shocks and reductions in farm income, water scarcity, and the concentration of household wealth in agriculture [26, 55, 65–67, 69–71]. These factors created additional stresses, exacerbated existing power imbalances, and contributed to the increased prevalence of GBV.

Some of these reported aggravating factors were disputed by other papers included in the review. Cooper et al. [72], for example, explored the association between intimate partner violence and drought and found no significant relationship. However, it is important to note that this research focused on regions such as Africa, the Americas, and Asia, leaving room for further investigation in other LMICs. Additionally, the work status of women was examined in relation to protection from GBV. Zafar et al. [73] conducted a study comparing an association between a woman’s work status in agriculture versus blue-collar or white-collar work and protection from GBV. Interestingly, their findings did not show a significant association between work status and protection from GBV. These results challenged the notion that a woman’s occupation alone can serve as a protective factor against GBV. These discrepancies highlight the complexity of aggravating factors influencing GBV, and the importance of conducting further research.

Protective factors that contributed to mitigating GBV merit further attention. These factors encompassed various aspects, including socioeconomic characteristics, household income, and some features of development programs. Examples of such factors include women’s ownership of land and homes, independent income, access to production-related natural resources, involvement in agricultural projects, and higher levels of education [25, 46, 53, 62, 65]. Additionally, women themselves adopted protective measures, such as working away from abusive partners [53]. The influence of culturally appropriate and context specific development programs, training initiatives, and non-governmental organizations (NGOs) also played a protective role. For instance, in Papua New Guinea, men showed more support for women’s involvement in beekeeping when they were engaged from the start of the program [52]. In Brazil, however, women revealed resistance to the presence of men in agricultural training, as it hindered willingness to share their perspectives and experiences, including those related to GBV [39]. Women-only spaces were viewed as opportunities for shaping an “agroecological popular feminist identity” and emancipation from oppressive social structures (38). These discrepancies highlight the need for interventions that are tailored to the specific cultural context.

### GBV interventions

Our review identified seventeen (35.42%) peer-reviewed journal articles that discussed primary and secondary prevention interventions. Primary prevention interventions focused on addressing the root causes of GBV to prevent violence from occurring; secondary interventions involved early intervention and measures to identify and respond to GBV incidents promptly [74].

Various primary prevention interventions were implemented to address GBV in agricultural settings. These diverse interventions aimed to create safer environments and promote gender equality in farming communities. Examples of interventions included: the prevention of GBV as a key demand in the collective bargaining agreements of agricultural workers to create supportive and respectful work environments [50]; poverty alleviation programs to address economic disparities and vulnerabilities that can contribute to violence [75]; to include gender mainstreaming within food security programs [76]; and interventions such as cash transfers, provision of supplies and targeted income support to address the underlying stressors that can fuel GBV [18, 70]. Engaging women in agricultural programs that increase food access, improve natural resource management and reduce family stress was also identified as an effective intervention [66, 69, 70, 77]. By empowering women and promoting their active participation in agricultural activities, these programs enhanced their economic independence and contributed to more equitable and harmonious family dynamics.

Integrated community-based education and economic empowerment programs were identified as potentially effective primary prevention approaches to addressing GBV. By addressing attitudes, beliefs, and behaviours that perpetuate violence, these programs focused on transforming social norms and promoting positive masculinity [66, 78]. Simultaneously, such programs provided opportunities for economic empowerment, giving individuals the means to thrive and reducing their vulnerability to GBV [18]. Gender dialogue groups involving women and their male partners were also implemented as part of economic empowerment programs. These groups provided a platform for open discussions on gender roles, norms and power dynamics, facilitating a deeper understanding and encouraging joint efforts to challenge and overcome GBV [78].

Research in Cambodia, for example, highlighted the role of economic development and primary production training programs in fostering increased recognition of gender equality [61]. Agricultural colleges also played a role in addressing GBV by delivering interventions and staff training to address GBV issues [79, 80]. By incorporating GBV education and awareness into the curriculum, and by using innovative approaches to address GBV, for example through “serious games” which included digital storytelling involving action characters and avatars, colleges were able to shape the attitudes and behaviours of future agricultural professionals and foster a culture of respect and equality [79, 80]. Finally, the promotion of ‘agrofeminist’ movements which sought to address gender inequality and promote women’s rights and agency, contributed to raising awareness of the unique challenges faced by women producers [81].

While primary prevention strategies focus on preventing GBV before it occurs, secondary prevention strategies are equally crucial to respond to the experiences and impacts of violence. Integrated approaches that addressed GBV in agricultural communities were crucial for fostering safer and more equitable environments [18, 50, 66, 69, 70, 75, 79, 80, 82]. In the context of addressing GBV in the agricultural sector, the emphasis on secondary prevention measures was relatively limited. However, interventions such as couple mediation, conflict resolution, and programs targeting husbands and fathers did demonstrate positive impacts on promoting healthier relationships [66, 79, 82]. These initiatives sought to engage men as allies in the fight against GBV and encouraged them to become active participants in fostering gender equality [78]. To ensure the effectiveness of interventions, a case-by-case approach to the interaction and engagement of both men and women was highlighted [63, 83]. Authors also highlighted the importance of developing context specific programs that address the social norms perpetuating GBV and aim to dispel the stigma associated with this issue [18, 66, 73, 80]. The importance of thoroughly evaluating prevention strategies, both within and beyond the intervention period, was also highlighted [18, 55].

## Discussion

As this scoping review reveals, there is a paucity in current state of knowledge regarding GBV in the context of women primary producers in LMICs. With just 49 relevant published research studies in the period from 2012 to June 2022, the body of research is not commensurate with the magnitude of the issue of GBV. Several authors have attempted to address the knowledge gap on GBV in agriculture, however, they face challenges due to under-reporting of GBV among women primary producers. Under reporting of GBV occurs worldwide and is not limited to LMICs [84, 85]. As such, the true extent of GBV perpetrated against women globally is far from fully understood. To gain sufficient insights into the extent of the challenge of GBV faced by women primary producers in LMICs, strategies for capturing data on GBV must be improved. Contextual research to better understand the reasons for under reporting is being undertaken [86, 87]. Furthermore, novel data collection approaches to capture prevalence data, including methods that increase respondent privacy and anonymity, are being trialed worldwide [84, 88]. Recent research from HICs underscores the crucial role of healthcare professionals in recognising and responding to GBV [89, 90]. These studies show promising outcomes, and highlight the importance of creating private, secure, and supportive settings where women can feel at ease to disclose and possibly report such incidents. Scant as the body of research may be, our scoping review did reveal that the forms and nature of GBV in agricultural settings is varied and complex. Our scoping review was, therefore, able to identify several research gaps and recommendations for future investigation.

Our findings highlight the limited focus on exploring and reporting women’s experiences of violence. The issue of under-reporting of GBV experiences among women is significant, contributing to a limited understanding of the nature of experiences and the likelihood of its impacts on women’s everyday life [2]. Efforts to capture the nature and extent of GBV have been made, employing quantitative survey approaches and tools like the Women’s Empowerment in Agriculture Index (WEAI) [91]. However, these approaches sit uneasily alongside feminist critiques of quantitative approaches in adequately capturing women’s lived experiences [92]. Our findings support the use of qualitative approaches to better understand the unique experiences of women, and the complexities of violence within specific cultural and contextual settings [16, 92]. By centering the voices and experiences of women producers who have lived through GBV, lived experience research empowers women to share their stories and ensures their perspectives are heard.

Our findings also draw attention to the limited scholarship on psychological forms of GBV in this setting. Women who are exposed to ongoing psychological abuse often suffer from long-term chronic mental health issues [93]. Unfortunately, most cases are treated without recognising GBV as a factor influencing mental health and overall well-being [94]. The limited data on this issue may be attributed to local understandings of what constitutes GBV; research that explores the cultural context and local understandings of GBV is warranted. Future research could also focus on psychological forms of GBV and how this intersects with other social and environmental factors that come into play in production settings. More specifically, survivor-led, trauma informed research approaches which draw upon women’s diverse and intersectional lived experiences [95, 96] are suggested to explore the complex and lasting impacts of GBV on women producers.

The influence of underlying social, cultural, economic and political factors leading to various forms of GBV have also been highlighted. In line with research undertaken by Hatcher et al. [6], we argue that more context specific research is required to better understand the extent and impact of GBV on women producers in LMICs. Power inequalities have historically been a significant feature of research in LMICs [97]. As noted by Thomas et al. [98], the power differentials are amplified further in the context of GBV research, with gender-based structural and cultural inequalities perpetuating the conditions in which violence can occur.

Participatory research approaches in tandem with meaningful research partnerships, offer a potential route to explore the issue of GBV in agricultural settings. Approaches such as Community Based Participatory Research (CBPR) [99], enable local research capacity and seek to redress the power imbalance that underpin violence and gender inequalities [95, 98]. We also argue for inclusive research approaches that incorporate the voices and lived experiences of women and men from diverse social groups. Research undertaken by CGIAR [100] to explore how gender norms affect, access and benefit from agricultural innovation, also notes the importance of participatory research approaches which gather contextually grounded evidence from “locals who crosscut society groupings”.

Our findings highlight the influence of deeply rooted patriarchy within many LMICs, where prevailing models of masculinity typically normalize violence as a legitimate means of resolving conflict or expressing anger, and where GBV is often dismissed [101]. In many LMICs, women producers face limited power in decisions about production, lack access to resources, and experience a lack of control over household incomes derived from agricultural activities [102]. Despite the submissive status of women in most LMICs, women bear responsibility for feeding their families and ensuring household food security [103]. This creates a critical paradox where women with limited access to decision-making, resources, and finances face the highest pressure to increase productivity to sustain the income required to manage the everyday essentials for their families.

In LMICS, women’s marginalised status is intensified by the consistent threat of violence if they fail to fulfill their sanctioned roles in the family and society. With the direct link between experiences of GBV, women’s capacity to participate in social and economic activities, and their sense of wellbeing, it remains critical to highlight the influence of patriarchal systems that further restrict women’s agency [104]. Future qualitative research could explore how deeply rooted patriarchy and rights to inheritance, such as land rights, contribute to gender-based violence in agriculture in LMICs. Research could also explore how cultural norms, traditions, and patriarchal systems restrict women producers from having ownership and control of their lives in LMICs. Importantly, men’s involvement in research is critical in addressing the gendered dynamics around GBV; men’s involvement demonstrates solidarity and shared responsibility in addressing GBV [16, 105]. Identifying ways in which men as well as women in LMICs can be safely engaged in future research, is an important step.

Our review also notes that, to identify effective interventions, an understanding of the context in which GBV occurs is needed. A blanket approach to developing interventions risks being ineffective, or worse, exacerbating the risks for women and girls [16, 92]; so context specific and culturally responsive approaches are necessary to address GBV in LMICs. Just as the factors that lead to GBV are complex, so too are the measures needed to devise culturally effective interventions for reducing GBV [4, 5]. A range of intersecting factors, such as coping strategies used by women primary producers, historical or traditional foundations of gender norms, government policy, the efficacy of the justice system, education, economic situation, resource availability, marital customs, and land inheritance customs, contribute to the complexity of developing effective GBV interventions. There is a need, therefore, to approach these intersecting factors at a fundamental level. Future research should focus on how to develop a comprehensive framework to guide the design and implementation of GBV interventions in agriculture, considering the intersecting factors and complexities involved. Culturally appropriate and gender-sensitive monitoring indicators of interventions on men and women are also an important area for future research.

Finally, the interaction between climate change and GBV in agricultural settings in LMICs is a significant gap in the literature. While some studies have highlighted the link between climate change and GBV risk [67, 71, 72, 77], the impact of climate change on primary production and its potential to exacerbate GBV risk remains unclear in the peer-reviewed literature. Although, there is increasing recognition that in some contexts, women in agriculture and food systems are more impacted by the adverse effects of climate change compared to men [106], further investigation into the complex interplay between climate change and GBV is warranted.

Limitations.

Although this scoping reviewed 49 articles and provides a baseline understanding regarding GBV in the context of women primary producers in LMICs, the review had several limitations. Firstly, the inclusion criteria were restricted to studies published in English in a peer-reviewed journal. We may have missed critical points published in the grey literature or presented in another language, however, given the wide range of results from quality studies, it is unlikely that significant findings were missed. The use of scoping review methodology is a comprehensive, rigorous, and a well-applied method of searching evidence [31], however, the review did not include a critical appraisal of the studies included, which may have limited the ability to assess the validity and reliability of the findings. Using multiple reviewers at each stage of review, and inclusion criteria forms added strength to the review.

The effectiveness of search terms in scoping reviews is crucial for ensuring a comprehensive range of relevant studies is included in the analysis. It is important, therefore, to acknowledge the inherent limitations of relying solely on specific keywords to capture the breadth of a particular field, especially in a context where culturally aligned terms exist. In regions such as Melanesia, for example, small area vegetable and fruit gardening systems, may constitute a predominant form of agriculture, but may not be referred to as such. The narrow scope of search terms such as “agriculture” or “farming”, for example, may have inadvertently excluded such studies from the review. Moreover, the scoping review was also challenged by different meanings and definitions of GBV. Depending on the specific definition used, certain studies or aspects of GBV may have been excluded, leading to a narrower scope. While the authors considered various terms and synonyms associated with GBV as part of the search strategy, the different meanings and definitions posed challenges in terms of the scope, comparability, and interpretation of findings.

## Conclusion

Many articles in our scoping review were able to articulate the often-unique challenges faced by women primary producers or women who live in agricultural/rural settings with regards to GBV. While studies varied in their findings on some of the interactions between GBV and agricultural settings, like the role of climate change and the impact of involving men in interventions, common threads bound the body of research together. These included the role of sociocultural factors in GBV, the impact of GBV on women’s agricultural livelihood activities and contemporary or historical aspects of primary production that shape attitudes toward GBV. The links between patterns or attitudes of GBV and the agricultural context was a common feature of study findings and informed recommendations for future research or interventions.

Our findings call for more qualitative and participatory research approaches to better understand the unique experiences of women and the complexities of violence within specific cultural and contextual settings. Our findings also highlight the limited scholarship on psychological forms of GBV in this setting, and the need for survivor-led, trauma-informed research approaches which draw upon women’s diverse and intersectional lived experiences. The importance of understanding the sociocultural and economic context in which GBV occurs to identify effective interventions, and the need for culturally appropriate and gender-sensitive indicators that can monitor the impact of interventions on men and women is required. Finally, our findings highlight the influence of deeply rooted patriarchy within many LMICs, where prevailing models of masculinity typically normalize violence as a legitimate means of resolving conflict or expressing anger, and where GBV is often dismissed. Future research needs to explore how deeply rooted patriarchy contributes to gender-based violence in agriculture in LMICs, and importantly involve both men and women in the dialogue.

## Data Availability

The datasets used and/or analysed during the current study are available from the corresponding author on reasonable request.
